# Efficacy of self-management program associated with a spa therapy for knee osteoarthritis patients (GETT 2): a research protocol for a randomized trial

**DOI:** 10.1186/s13063-022-06879-5

**Published:** 2023-01-19

**Authors:** A. Goldstein, C. Lanhers, C. Gay, K. Dubourg, L. Grange, C. F. Roques, B. Pereira, E. Coudeyre

**Affiliations:** 1 Service de Médecine Physique et de Réadaptation, CHU Clermont-Ferrand, Université Clermont Auvergne, INRAE, UNH, F-63000 Clermont–Ferrand, France; 2 Service de Santé Publique, CHU de Clermont Ferrand, PEPRADE, Université Clermont Auvergne, Clermont–Ferrand, France; 3 Université de Bordeaux, Institut du Thermalisme, Dax, France; 4 Service de Rhumatologie, C.H.U.G.A Hôpital Sud, Échirolles, France; 5 AFRETH, Paris, France; 6 Délégation Recherche Clinique et Innovation, CHU de Clermont Ferrand, Université Clermont Auvergne, Clermont–Ferrand, France

**Keywords:** Knee osteoarthritis, Patients education, Spa-therapy, Self-management, Physical activity, Randomized control trial

## Abstract

**Introduction:**

Osteoarthritis is a chronic pathology that involves multidisciplinary management. Self-management for patients is an essential element, present in all international guidelines. During the time of the spa therapy, the patient is receptive to take the advantage of self-management workshops.

The aim of this study is to assess the effects of 18 days spa therapy associated with a self-management intervention in patients with knee osteoarthritis in comparison with spa therapy alone on a priority objective, personalized and determined with the patient, chosen in the list of 5 objectives determined during the self-management initial assessment.

**Methods and analysis:**

Two hundred fifty participants with knee osteoarthritis will participate to this multicenter, prospective, randomized, controlled study. All patients will benefit 18 days of spa therapy and patients randomized in the intervention group will participate to 6 self-management workshops. Randomization will be centralized. The allocation ratio will be 1:1. Data analysts and assessor will be blinded. The primary outcome is the effectiveness of the educational workshops associated with spa therapy in comparison with spa therapy alone on a priority objective, measured by Goal Attainment Scaling (GAS). The secondary outcomes are disability, health-related quality of life, and pain intensity.

**Ethics and dissemination:**

Ethics were approved by the CPP *Sud-Méditerranée II*. The results will be disseminated in a peer-reviewed journal and disseminated at PRM, rheumatology, and orthopedics conferences. The results will also be disseminated to patients.

**Trial registration:**

Trial registration number NCT03550547. Registered 8 June 2018.

Date and version identifier of the protocol. Version N°6 of March 12, 2018.

## Introduction

Osteoarthritis (OA) is the most common diagnosis made by general practitioners in older patients, and OA is the most common arthropathy to affect the knee [1]. About 25% of adults aged > 55 years experience significant knee pain; half of these have radiographic changes of OA and a quarter have significant disability [2]. According to the World Health Organization (WHO), in 2020, chronic disease will be the main source of disability. This evolution is related to the increase in life expectancy due to improvements in medical technology [3]. Risk factors are multiple: heredity, overweight, and trauma (sports, professional and surgery) [4, 5]. All these factors interact with each other and may contribute to worsened pain and disability and reduced mobility. According to the first evaluation of the socioeconomic impact of osteoarthritis in France conducted in 1993, the estimated annual cost was slightly less than one billion euros [6]. In 2003, the annual cost was approximately 1.8 billion euros. Overall costs for osteoarthritis have thus increased by more than 80%, i.e. 8% per year [7].

With lack of any curative treatment, except prosthetic surgery, non-pharmacological treatment is essential [8]. OA is a chronic joint disease that involves multidisciplinary care. International guidelines such as from the Osteoarthritis Research Society International (OARSI) recommend a non-pharmacological intervention associated with pharmacological treatment for pain for people with knee OA. The self-management of the patient is an essential element in all international recommendations.

Non-pharmacological therapies are exercise programs, self-management education program, and weight loss if necessary [9–11]. Education and self-management have a positive impact on pain, function, exercise level, weight, quality of life, and treatment adherence [12].

The efficiency of spa therapy was also demonstrated in knee OA [13]. Mechanisms of action in spa therapy treatment are not fully understood but a combination of factors: mechanical, thermal, and chemical seems to be the most evident [14]. Spa therapy resorts, common in Europe, include a large sample of patients with different phenotypes from early to advanced OA stages. The spa treatment context could offer good conditions for behavioral modification and could be a special opportunity for self-management [15].

On the other hand, the synergic effect of a self-management program associated with spa therapy for the patients with OA is still debated The spa resort is the opportunity to meet and interact with other patients and benefit from multidisciplinary medical and paramedical support for ameliorating pain and disability [16].

The proposed study will address to the persons having spa therapy for OA. It will be a question of proposing to them a spa therapy of 18 days associated with a self-management intervention on a priority objective, personalized and determined with the patient, chosen in the list of 5 objectives determined during the educational initial assessment.

We aim to conduct a multicenter, prospective, randomized study to evaluate the effectiveness of a 6 personalized self-management workshops associated with a spa therapy journey of 18 days. The proposed educational activities will focus on key areas of leadership in the OA: knowledge and beliefs of the pathology, educational physical exercise, diet, management of pain, articular ergonomics, and medical devices. The objective looked for by these programs is a real modification of the lifestyle of the patients.

This study is aimed at people with knee OA. The main objective is to measure the impact of 18 days spa therapy associated with an educational intervention in patients with knee osteoarthritis on a priority objective, personalized and determined by the patient, chosen in the list of 5 objectives determined during the educational assessment.

The secondary objectives are to compare the groups in terms of pain, disability, and health-related quality of life.

## Methods

### Trial design

This is a multicenter, prospective, comparative, superiority randomized trial. The population will be randomized in 2 arms: experimental group of a 6 personalized self-management workshops associated with a spa therapy journey of 18 days versus spa therapy of 18 days alone (active comparator).

The design and conduct of this trial will adhere to the requirements of the Standard Protocol Items: Recommendations for Interventional Trials (SPIRIT Annex). The results will be reported in accordance with the CONSORT Statement for non-pharmacologic trials.

### Participants

We will recruit 250 participants, male and female, who are 50–75 years old with a diagnosis of mono or bilateral knee OA, in 6 spa therapy resorts, in France. All people already registered for STT will receive an information letter with study notification and eligibility criteria. The center is Clermont-Ferrand University Hospital associated with Dax and Saint-Paul-Lès-Dax spa center, Royat, Bourbon-Lancy, Chaudes Aigues, Aix-les-Bains,Evaux les Bains, and Balaruc spa centers. Patient recruitment potential among people with knee OA in spa therapy is important. Indeed, OA represents the main disease treated by spa therapy (250,000 per year in France). For people who meet the inclusion criteria, the research coordinator will perform the information and consent process and the physician will verify the inclusion criteria. The full description of the eligibility criteria for participants is provided in Table 1.

**Table 1:** Eligibility criteria for participants

Inclusion criteria	− People, male or female, 50 to 75 years old − Mono or bilateral knee OA according to American College of Rheumatology (ACR) criteria − Pain on an 11-point NRS ≥ 30/100 − Written consent obtained − Health insurance cover
Exclusion criteria	− Contraindication to spa therapy − Unstable angina − Cardiac failure − Behavioral disorders or comprehension difficulties making assessment impossible − Inability to speak, write, or read French language

### Randomization and allocation concealment

Participants who meet inclusion criteria and agree to participate will be randomly assigned (1:1) to the experimental or comparator group using the REDCap software. The randomization sequence will be computer-generated by an independent statistician using permuted variable block sizes stratified by centers. The randomization process will be centralized at the coordinating office which will have no involvement in the enrollment, follow-up, or assessment of participants. Only the independent statistician have access to the randomization list and allocation concealment.

Access to the system is controlled for each investigator by an individual login/password and using a secured https connection. Participants also have paper files which are anonymized and only contain the participant’s unique identification code. These are stored in a dedicated storage unit in each center. Access to the complete final trial dataset will be restricted to the statistician of the *Secteur Biométrie et Médico-économie* who will analyze the study data for the purpose of report and publication.

### Interventions

Both groups will receive a spa therapy during 18 days. Only the experimental group will receive the personalized self-management workshops. The aim is to propose a reinforcement of the spa therapy effect through 6 self-management workshops. The STT and the self-management workshops will be personalized and designed by expert spa therapy physicians. Interventions have been designed by a specialist steering committee, standardized and reproducible for all centers. The steering committee consists of investigators, a spa therapist, physician, and physiotherapist and the university hospital’s medical team (physician, physiotherapist, adapted physical activity instructor). Self-management program has been implemented since 2018 in Dax thermal care facilities. It aims to make the patients able to improve their quality of life by an accurate comprehension of their particular situation and participation in six workshops dedicated to get the necessary skills. The workshops aim at better understanding of the disease, use of drug treatments, nutrition, joint protection, and physical activity. The self-management program has been patronized by the French Association for Thermal Research (AFRETh) and was agreed by Nouvelle Aquitaine Regional Health Agency (ARS). A personalized educational diagnosis allows the identification of personal goals to be achieved by the patient.

Meetings with all members of the steering committee are organized to obtain consensus on the therapeutic protocol describing precisely the content and the organization of the intervention taking into account the context and the expertise of the different centers involved in the study.

**Fig. 1:**
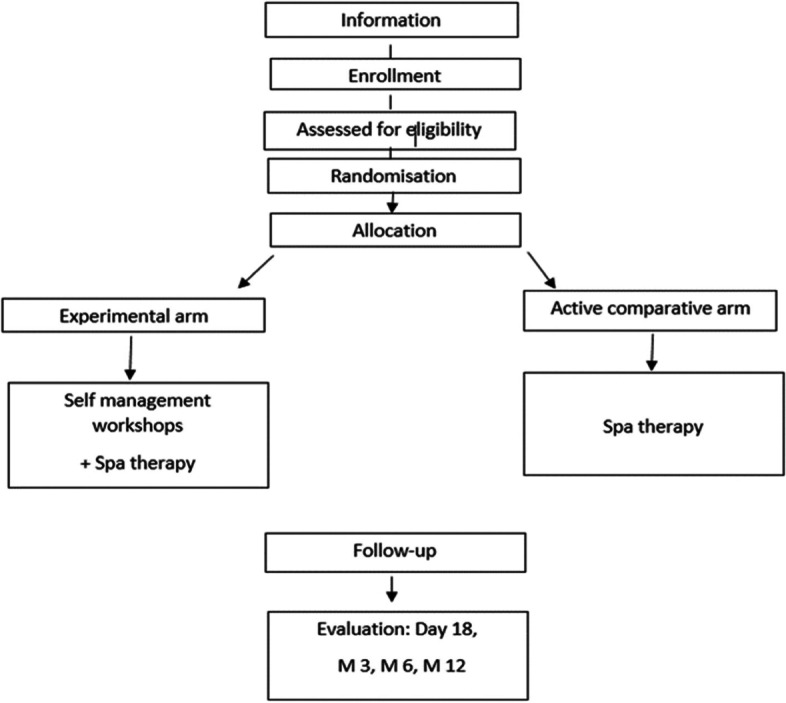
Flow of the participants through the study

A flow of the participants through the study is provided in Fig. 1.

The Data Monitoring Committee (DMC) will be responsible for safeguarding the interests of trial participants, assessing the safety and efficacy of the interventions during the trial, and for monitoring the overall conduct of the clinical trial. The DMC will provide recommendations about stopping or continuing the trial to the Steering Committee (SC) of the trial. The SC will be responsible for promptly reviewing the DMC recommendations, to decide whether to continue or terminate the trial and to determine whether amendments to the protocol or changes in trial conduct are required.

The DMSC is an independent multidisciplinary group consisting of clinicians and a biostatistician that, collectively, have experience in the conduct, monitoring and analysis of randomized clinical trials. The members of the DMSC will be chosen among experts without conflicts of interest that could be perceived as inferring with the study.

This independent DMC will meet a first time at study initiation and then throughout the duration of the study at its own initiative or at the sponsor’s request and after 50% of inclusions.

The DMC will remain blinded for the allocation during analysis; however, the observation of differences in serious adverse events between the two groups may allow, for safety reasons, to unblind allocation groups.

### Self-care workshops

The main objective of the health education workshops will be to enable patients to acquire knowledge and competencies (attitudes, behavior) regarding their pathology and existing treatments. Each of the 6 health educational workshops will last between 1 h and 1 h and a half (Table 2). A multidisciplinary team will assure the educational interventions. To facilitate the workshops, different teaching tools and techniques will be used: paper board, thematic discussion, participative discussion, practical leaflets (Borg scale, deconditioning circle), exercise demonstration, feedback, and pain coping skills. The exchanges between the patients and the educative personals as well as the motivational communication will be the basic principles for every workshop.

**Table 2:** Predefined objectives for each self-management workshop

Workshops	Objectives
Knowledge of the pathology	− Know the mechanisms of the OA and its risk factors, evolutionary modes and main current therapeutic strategies − Be able to speak usefully about OA and identify situations where it is necessary to see a doctor
Educational physical exercise practice	− Highlight the interest of the Adapted Physical Activity − Pacification and adaptation of physical activity to progress by knowing how to recognize the physical potential and the limits
Diet	− Diet in relation to the presence of knee osteoarthritis
Management of pain, fatigue, and the medical treatments	− Identify the relationship with the pharmacological treatments − Describe the non-pharmacological treatments for OA: psychological and physical
Articular ergonomics	− Allow for joint protection in everyday life − Better move by mobilizing the joint and protecting it during the movement.
Medical devices, an adaptation to the living conditions	− Foot orthoses, knee braces and technical helps.

### SPA therapy treatment

Each spa therapy session will comprise a mineral hydrojet session at 37 °C for 15 min, the thigh manipulated under mineral water at 38 °C by a physiotherapist for 10 min, manual massages, the application of mineral matured mud at 45 °C to the knees for 15 min, and supervised general mobilization in a collective mineral water pool at 32 °C in groups of 8 patients for 15 min of 18 sessions, for 1 h each. Both groups will be mixed with the general public in the spa center. The spa therapist will not be aware of which patients will be taking part in the clinical trial. The experimental and active comparator arms will receive unrestricted non-pharmacological, pharmacological, and usual care during the study. Medication taken during or after the intervention will be reported in the follow-up questionnaires.

### Measures

At baseline, we will collect data on sociodemographic items (age, marital status, area living, education status), anthropometric measures, and co-morbidities association, according to OARSI guidelines.

### Primary outcomes

The primary outcome is change from effectiveness of the educational workshops, personalized and determined for every patient, measured by a GAS (Goal Attainment Scaling in Rehabilitation) [17] at 6 months, method of scoring the extent to which patient’s individual goals are achieved in the course of intervention. In effect, each patient has their own outcome measure but this is scored in a standardized way as to allow statistical analysis. Each goal is rated on a 5-point scale, with the degree of attainment captured for each goal area:

- If the patient achieves the expected level, this is scored at 0.
- If they achieve a better than expected outcome, this is scored at + 1 (more than expected), + 2 (much more than expected)
- If they achieve a worse than expected outcome, this is scored at − 1 (less than expected) or − 2 (much less than expected).

GAS depends on two things—the patient’s ability to achieve their goals and the clinician’s ability to predict outcome, which requires knowledge and experience. The process of the setting of the goals will be realized during the Initial educational assessment for each patient by specially trained nurses. For each patient, the main problem areas will be identify and establish an agreed set of priority goal areas (in connection with the themes of the workshops). Set goals should follow the SMART principle: they should be Specific, Measurable, Attainable, Realistic and Timely. GAS will be collected by clinical research fellow dedicated to this task that will be blinded.

### Secondary outcomes

- Physical function is assessed by the Western and McMaster Universities Osteoarthritis Index (WOMAC subscale for physical function (W-TPFS)).
- Pain intensity during the last 24 h and the worst pain intensity during the last month are measured by a measured on a visual analog scale (VAS).
- Health-related quality of life (HRQOF) measured by the SF36, one of the most widely used generic measures of health-related quality of life.
- Comprehensive evaluation of patient education programs measured by a HEIQ. (Health Education Impact Questionnaire), an outcome and evaluation measure for patient education and self-management interventions for people with chronic conditions.

All the secondary outcomes will be collected at 6 months**.**

### Statistical considerations

#### Sample size estimation

According to the previous works presented in literature, we have estimated that a sample size of 105 patients per randomized group would provide 95% statistical power of highlighting an effect-size greater than 0.5 for a two-sided type I error at 5% for the primary outcome GAS T-score. Finally, a total of 250 patients (125 by group) will be considered to take into account lost to follow-up. With such sample size, a minimal absolute difference of 20% (50% versus 30%) can be shown for the secondary endpoint clinical improvement as defined in the thermarthrose [13] study for a power of 90%.

#### Statistical analysis

Statistical analyses will be conducted using the Stata software (version 13, StataCorp, College Station, USA). A two-sided *p*-value of less than 5% will be considered to indicate statistical significance.

Concerning the primary outcome, the comparison between groups for univariate analysis will be analyzed using Student’s *t*-test or Mann-Whitney’s test. The normality will be studied by the Shapiro-Wilk test and the homoscedasticity using the Fisher-Snedecor test. Then, the analysis of the primary outcome will be completed by multivariable analysis using a linear mixed model to take into account: (1) fixed effects covariates determined according to univariate results and to clinical relevance (for example gender, age, analgesic treatments and season) and (2) center as random-effects (to measure between and within center variability). The normality of residuals will be studied as aforementioned. Intention-to-treat (ITT) analysis will be considered for the primary outcome, more precisely for all randomized patients except those without consent.

Other continuous endpoints (WOMAC, HEIQ, pain VAS, quality of life SF36, BMI) will be studied using the same statistical plan whereas the categorical parameters will be compared between groups with chi-squared or Fisher’s exact tests in univariate analyses and using generalized linear mixed model for multivariable analyses. For HEIQ and SF36 scores and pain VAS, the comparisons between groups will include in multivariable analyses the baseline values as independent parameters (fixed effects) as suggested by Vickers and Altman [18].

Longitudinal analyses concerning repeated measures at day 18 and months 3, 6, and 12 will be studied using random-effect models (linear or generalized linear), to take into account patient as random-effect (slope and intercept), nestled in center random-effect, while studying the fixed effects: group, time, and interaction *group x time*. According to clinical relevance, sub-group analyses depending the gender will be proposed after the analysis of interaction sub-group x randomization group in regression models (for repeated data or not).

Secondarily, a per-protocol analysis will be considered. A particular attention will be paid on number and duration of sessions and the type of intervention.

Finally, a sensitivity analysis will be performed and the nature of missing data will be studied (missing at random or not). According to this, the most appropriate approach to the imputation of missing data will be proposed (maximum bias (e.g., last observation carried forward vs. baseline observation carried forward) or estimation proposed by Verbeke and Molenberghs for repeated data).

#### Ethics

All patients will receive verbal and written information on the aim of the study and the protocol. Written informed consent will be obtained prior to their inclusion in the study and before performing any specific procedure. During the study, patient will have the opportunity to ask all questions concerning the protocol to the investigator. They will be informed that they are free to stop the study at any time at their own discretion, in accordance with the Good Clinical Practice in current enforced under the French regulatory framework. Consent form is available from the corresponding author on request.

Any adverse event or serious adverse event that could occur during the protocol will be reported to the DMC and relevant regulatory bodies indicating expectedness, seriousness, severity, and causality. Should there be any negative impact of participating in the study on the patient’s health status, the participant will be entitled to compensation in accordance with the French regulations.

Pursuant to the provisions concerning the confidentiality of data that are available to persons responsible for quality control of biomedical research, persons with direct access will take all necessary precautions to ensure the confidentiality of information (identity and patients results). Data collected will be made anonymous.

Any changes in the protocol will be notified to the AFRETH then the primary investigator will notify the centers. A copy of the revised protocol will be sent to the primary investigator to add to the Investigator Site File. Any deviations from the protocol will be fully documented using a breach report form and will update the protocol in the Clinical Trials website.

On the consent form, participants will be asked if they agree to use of their data should they choose to withdraw from the trial. Participants will also be asked for permission for the research team to share relevant data with people from the universities taking part in the research or from regulatory authorities, where relevant. This trial does not involve collecting biological specimens for storage.

#### Patient and public involvement

Patients were not involved in the development and the design of the study. The burden of the intervention will not be assessed by patients themselves. Patients will receive a written summary of tests and evaluation results they will have completed during their rehabilitation and will be written informed of global study results at its end.

#### Management of the study

Data will be collected and managed using REDCap (Research Electronic Data Capture) electronic data capture tools hosted at the University Hospital of Clermont-Ferrand. REDCap is a secure, web-based application designed to support data capture for research studies, providing:

- An intuitive interface for validated data entry;
- Audit trails for tracking data manipulation and export procedures;
- Automated export procedures for seamless data downloads to common statistical packages;
- Procedures for importing data from external sources.

A clinical research assistant will be commissioned to ensure that the progress of the study and the data are captured according to the Standard Operating Procedures implemented at the University Hospital of Clermont-Ferrand.

The trial steering committee consists of the sponsor, the investigator, and the researcher responsible for the study. The trial steering committee meets every second month to ensure that the trial conforms to the protocol and are also available to the clinicians for day to day support and questions regarding the trial and participants. Additionally, two professors, who are independent from the data collection, are continuously supervising the trial with yearly meetings with the trial steering committee and will supervise the data analysis and interpretation of data.

K Dubourg and the nurses in charge of the study will be responsible for all aspects of local organization including identifying potential recruits and taking consent.

#### Data monitoring

Quality control and quality assurance follows regular procedures. The internal monitoring team are independent from the intervention and have no competing interests. When a participant is recruited and obtains written informed consent, the monitoring team afterwards check that it is filled out correctly.

An interim analyses will be provided in case of insufficient recruitment of the study and if needed stopping the trial.

## Discussion

OA is a high-prevalence disease, whose prevalence will increase in the future [19]. Treatments will be based on modifiable risk factors, such as pain, function, obesity, comorbidities, intrinsic barriers to PA practice, and sedentary time by the real modification of the lifestyle of the patients [20, 21].

This randomized trial will be the first study to compare the effect of a self-management workshops associated with spa therapy versus spa therapy alone. The efficacy of spa therapy in knee OA has been demonstrated, with good level of evidence for pain and disability [22], but the effect of an additional self-management workshops is unknown. Our study would be the first to evaluate it and the main outcome will be measured by Goal Attainment Scaling (GAS).

Measurement through Goal Attainment Scaling (GAS) apply in many other areas including chronic pain [23], elderly care settings [24, 25], cognitive rehabilitation [26], and amputee rehabilitation [27]. GAS offers a number of potential advantages as an outcome measure. This goal-setting builds on already established process to encourage communication and collaboration between the multi-disciplinary team members as they meet together for goal-setting and scoring patient involvement—there is emerging evidence that goals are more likely to be achieved if patients are involved in setting them. Moreover, there is also evidence that GAS has positive therapeutic value in encouraging the patients to reach their goals [28].

In spite of the limitation, assessing the effectiveness of non-pharmacological treatments such as behavioral therapy the blinding of participants and care providers is frequently impossible; the success of the treatment often depends on the skill and experience of care providers. As an outcome measure, there is growing evidence for the sensitivity of GAS over standard measures [29]. It potentially avoids some of the problems of standardized measures including floor and ceiling effects and lack of sensitivity—particularly of global measures, where individuals make change in one or two important items, but this change is lost in the overall scores, where a large number of irrelevant items do not change [29]. One of the most attractive advantage of GAS is its individualized approach to evaluation.

The findings of this trial could offer new perspectives in establishing best clinical practice guidelines for this patient population.

## Trial status

The randomization of patients commenced in March 2019 and is ongoing till December 2023. Protocol Version: 6 – 12 March 2018.

## Data Availability

The datasets analyzed during the current study and statistical code are available from the corresponding author on reasonable request, as is the full protocol.
